# Measurement of the viscoelastic properties of blood plasma clot formation in response to tissue factor concentration-dependent activation

**DOI:** 10.1007/s00216-016-9689-3

**Published:** 2016-06-16

**Authors:** Ramji S. Lakshmanan, Vitaly Efremov, James S. O’Donnell, Anthony J. Killard

**Affiliations:** 1 Biomedical Diagnostics Institute (BDI), Dublin City University, Dublin 9, Ireland; 2 Haemostasis Research Group, Trinity College Dublin and National Centre for Hereditary Coagulation Disorders, St. James’s Hospital, Dublin 8, Ireland; 3 Centre for Research in Biosciences (CRIB), Department of Applied Sciences, University of the West of England, Coldharbour Lane, Bristol, BS16 1QY UK

**Keywords:** Blood, Coagulation, Viscoelasticity, QCM, Tissue factor, Rigidity factor

## Abstract

The coagulation of blood plasma in response to activation with a range of tissue factor (TF) concentrations was studied with a quartz crystal microbalance (QCM), where frequency and half width at half maximum (bandwidth) values measured from the conductance spectrum near resonant frequency were used. Continuous measurement of bandwidth along with the frequency allows for an understanding of the dissipative nature of the forming viscoelastic clot, thus providing information on the complex kinetics of the viscoelastic changes occurring during the clot formation process. Using a mathematical model, these changes in frequency and bandwidth have been used to derive novel QCM parameters of effective elasticity, effective mass density and rigidity factor of the viscoelastic layer. The responses of QCM were compared with those from thromboelastography (TEG) under identical conditions. It was demonstrated that the nature of the clot formed, as determined from the QCM parameters, was highly dependent on the rate of clot formation resulting from the TF concentration used for activation. These parameters could also be related to physical clot characteristics such as fibrin fibre diameter and fibre density, as determined by scanning electron microscopic image analysis. The maximum amplitude (MA) as measured by TEG, which purports to relate to clot strength, was unable to detect these differences.

## Introduction

Blood coagulation is a complex process involving several biochemical reactions leading to the formation of a clot, which is a gel-like network formed at a site of injury and which functions as a plug to prevent loss of blood. The coagulation cascade is triggered physiologically by tissue factor (TF) that is exposed on the sub-endothelium upon damage to the vessel wall. TF complexes with factor VII and leads to a series of reactions that result in the generation of thrombin. The enzymatic action of thrombin on the soluble protein fibrinogen present in blood produces fibrin monomers that assemble to form half-staggered oligomers that lengthen to form protofibrils [1–4], which eventually aggregate to form thicker fibres and branch out and grow laterally and longitudinally to form a three-dimensional network. They are also further cross-linked by covalent bonds formed by factor XIII. Kaolin and TF activation are commonly used for monitoring haemophilia [5–8]. However, TF is a physiological activator while kaolin is a non-physiological particulate activator. The physical properties of this network are largely dependent on the amount and rate at which thrombin acts on fibrinogen and is an important characteristic indicator of the quality of the clot [3, 4].

Global coagulation testing seeks to study all of the physical and biochemical processes taking place during clot formation, so as to better represent the physiological state. Clot formation results in unique time-dependent viscoelastic properties which are contingent on several factors that contribute to the coagulation process. Monitoring the blood coagulation process is important in understanding the haemostatic balance. The conventional “endpoint”-based assays only reveal one parameter of the time taken for the clot to achieve a threshold value. A global coagulation monitoring approach such as thromboelastography (TEG)—which is a viscoelastic method—allows the monitoring of the kinetics of the coagulation process and—in addition to providing the corresponding clot time values—provides an entire picture of viscoelastic changes occurring in the clot formation process. The TEG system has been widely used for over 40 years at point-of-care in operation rooms guiding clinicians with optimising transfusion management [9, 10]. Quartz crystal microbalance (QCM), a piezoelectric shear mode resonator, has the ability to measure coagulation endpoints along with coagulation kinetics [11–17]. QCM resonance is characterised by a resonant frequency (*f*) and bandwidth (half width at half maximum, *Γ*), both of which can be obtained using a Lorentzian curve fit of the conductance spectrum. When any material interacts with the QCM surface, monitoring *f* in association with *Γ* provides unique physical information relating the viscoelastic properties of the material. In previous work [11, 12], the effect of fibrinogen concentration on qualitative and quantitative viscoelastic changes during clot formation activated using a partial thromboplastin has been shown using QCM. In addition to measuring the activated partial thromboplastin time (aPTT), this method allowed the quantitative characterisation of fibrinogen concentrations as well as a qualitative characterisation of the properties of the forming clot in a single test [18], which usually requires two independent tests. Hussain et al. [17, 19] later reported a similar use of QCM in measuring clotting using the same aPTT assay for different fibrinogen concentrations. Since the method monitors the entire kinetics of the coagulation process, traditional parameters such as aPTT can be easily extracted. However, the main advantage of using QCM lies in the fact that it can also provide meaningful viscoelastic information on the forming clot.

An unloaded (in-air) QCM sensor is typically characterised by a high quality factor with a resonant frequency (*f*_air_) ≈5 MHz and half bandwidth (*Γ*_air_) ≤30 Hz. In this article, the mechanical properties of a TF-activated plasma sample loaded on the sensor were investigated with a mathematical model developed [18] by means of an effective elasticity (*μ*_*t*_),effective mass density (*M*_*t*_) and resulting rigidity factor (RF) calculated using the shifts of resonance peak characteristics, Δ*f* and Δ*Г*, with respect to their “in-air” values. Briefly, QCM is used to investigate the effect of TF activation for coagulation and show that the developed model relates differences in TF concentration to changes in the qualitative nature of the formed clot. Since this method continuously monitors the coagulation process, it allows information on clotting time, rate of clot formation and maximum amplitudes of both frequency and bandwidth changes to be obtained.

## Materials and methods

### Reagents

Water, calcium chloride (CaCl_2_), glutaraldehyde, ethanol, toluene and phosphate-buffered saline (PBS) were all obtained from Sigma-Aldrich. A Dade Innovin® reagent was used as the source of TF and was obtained from Sysmex. Vacutainer tubes (BD-367691) containing sodium citrate (0.105 M) and 21-gauge needles used for blood draw were obtained from Becton Dickinson.

### Preparation of platelet-poor plasma (PPP)

This study was approved by the Research Ethics Committee of Dublin City University. Informed consent was obtained from all volunteers prior to phlebotomy. Normal healthy controls were recruited at Dublin City University, and patients with cardiovascular disease or individuals taking anti-platelet medication (e.g. aspirin) were excluded from the study. Whole blood (WB) was obtained from normal donors using a 21-gauge needle by a trained phlebotomist into vacutainers containing sodium citrate. The vacutainers containing WB were centrifuged at 3000*g* for 10 min, and the top two thirds of the supernatant was carefully extracted to obtain PPP and was placed at 37 °C before use. Measurements were performed within 30 min of the blood draw.

### QCM sensor preparation and test methodology

The polished QCM sensor crystals with gold electrodes (Maxtek Inc., Torrance, CA, USA) were cleaned in piranha solution (H_2_SO_4_:H_2_O_2_ in 3:1 *v*/*v* ratio), followed by a thorough rinse in deionised water and dried in a stream of nitrogen. (*Caution*: piranha solution is highly corrosive and should be handled with care). A hydrophobic polystyrene (PS) layer was then spin-coated (2.7 % *w*/*w* PS in toluene; 2400 rpm for 1 min). The PS-coated sensors were cured in an oven at 60 °C for 1 h. The coated sensors were stored in vacuum desiccators until use and were typically used within 24 h of coating. After experiments conducted with plasma, QCM sensors were cleaned and reused. The remains of the insoluble fibrin clot were washed with copious amounts of water. This was followed by ultrasonic cleaning for 5 min each in 2 % (*w*/*v*) sodium dodecyl sulphate (SDS), deionised water and 70 % (*v*/*v*) ethanol, respectively. The PS coating was then dissolved from the surface using toluene. The resonant frequency and bandwidth were recorded for each sensor to ensure regeneration of the surface.

The experimental arrangement as described previously [11, 12] consisted of a QCM holder (SRS QCM200), a sample chamber and a network/spectrum/impedance analyser (Agilent, 4395A-010). The sample chamber was composed of Teflon® and was designed to hold 200 μL of the sample on top of the QCM, while preventing evaporation. The QCM resonant frequency characteristics (frequency (*f*) and bandwidth (*Γ*)) were determined by applying a rational fitting algorithm to the conductance spectrum near the QCM resonant frequency. QCM sensor electrodes were connected to the impedance analyser interfaced to a data acquisition PC running a custom LabVIEW® program that enabled collection of *f* and *Γ* measurements (bandwidth used was half width at half maximum). All QCM measurements were carried out in a Binder ED-53 oven (Bohemia, NY, USA) maintained at 37 °C.

### Coagulation measurements on QCM

The QCM was placed in the chamber and allowed to form a stable baseline frequency. The frequency shift was calculated as Δf^*^(*t*) = *f*(*t*) − Δ*f*_0_ and bandwidth shift as ΔΓ^*^(*t*) = *Γ*(*t*) − Δ*Г*_0_, where Δ*f*_0_ and Δ*Г*_0_ are corresponding Δ*f* and Δ*Г* values measured immediately after liquid sample loading, i.e. before the onset of coagulation. A 20-μL aliquot of the TF reagent (0.35, 1.42, 3.5, 14.2 and 35 pM in PBS) was added to a vial containing 200 μL of PPP, maintained at 37 °C and incubated for 5 min. Pre-warmed (37 °C) 20 μL of CaCl_2_ (100 mM) was added to this vial, and 200 μL of this mixture was immediately transferred to the QCM chamber. Time courses, Δf^*^(*t*) and ΔГ^*^(*t*), provide the information about the kinetics of clot formation, namely clotting time (R-QCM), maximum rate of clot formation (MRCF), time to maximum rate of clot formation (TMRCF) and maximum response (Δf^*^_max_ or ΔΓ^*^_max_). R-QCM was defined as the time taken from the start of activation to achieve 10 % of Δf^*^_max_. MRCF and TMRCF were obtained from the first derivative curves of the Δf^*^(*t*) or ΔΓ^*^(*t*) responses.

### TEG test methodology

The TEG tests were conducted with identical concentrations of TF reagent and CaCl_2_ concentrations for comparison of kinetics. The PPP, TF and 100 mM CaCl_2_ were pre-warmed to 37 °C on a heating block for 15 min. A 30-μL aliquot of the TF reagent (0.35, 1.42, 3.5, 14.2 and 35 pM) was added to a vial containing 300 μL of PPP, maintained at 37 °C and incubated for 5 min. Pre-warmed (37 °C) 30 μL of CaCl_2_ (100 mM) was added to the TEG cup followed by 330 μL of the PPP-TF mixture. TEG traces were recorded for a period of 30 min, and the corresponding reaction time (*R*-), time to maximum rate of thrombus generation (TMRTG), maximum rate of thrombus generation (MRTG) and maximum amplitude (MA) were recorded for comparison with QCM responses. In TEG, *R*- is defined as the time taken for the pin displacement to reach an amplitude of 2 mm from the start of activation.

### Scanning electron microscopy: sample preparation and image analysis

The samples used to image the clot formed on the sensor surface were prepared according to methods described elsewhere [20–22]. Briefly, the sensors were taken from the QCM holder and rinsed gently with deionised water and immediately placed in a 2.5 % (*v*/*v*) glutaraldehyde solution for 15 min. The sensors were then rinsed gently with deionised water several times. This was then followed by drying the sensors in increasing ethanol water mixtures of 20, 40, 60, 80 and 100 % (*v*/*v*) for 15 min each. The sensors were then dried in a stream of nitrogen, and a thin-conducting gold layer was sputter-coated to provide a conductive surface to perform SEM investigations.

SEM (accelerating voltage 5 kV) images at a magnification of 20,003 and 250,003 were obtained for plasma samples activated with three TF concentrations (0.35, 3.5 and 35 pM). In order to get numerical estimates for the fibre diameter and the fibre density, the lower-magnification (20,003) images were processed and analysed, as they contained representative ensembles of fibres and hence could provide statistically relevant values for the parameters of interest.

Image spectral analysis was applied as a series of processing steps [23]. In the first step, the image was filtered from the high-frequency structures (considered as noise) having a size of less than 2 pixels, which corresponds to less than 48 nm actual size. The result was converted to a binary image with a threshold chosen as the mean value of pixel intensity. Pixels with an original intensity less than threshold were given a value of −1 (“dark pixel”), and pixels with intensities higher than threshold were given a value of +1 (“bright pixel”). The average fibre network porosity, F.Por, was defined as the ratio of the number of dark pixels to the absolute number of image pixels. In the second step, a two-dimensional fast Fourier transform algorithm was applied to the binary image. The resulting matrix of spectral amplitudes was converted to a one-dimensional radially averaged power spectrum. The frequency value corresponding to maximal power was used to estimate the average fibre density (F.Dens). Finally, an estimate of average fibre diameter (F.Diam) was calculated from F.Por and F.Dens based on the assumption that the fibre network had an ideal form of rectangular gratings, that is F.Diam = (1 − sqrt(F.Por)) / F.Dens.

## Results and discussion

### Analysis of responses to activation with tissue factor

**Fig. 1:**
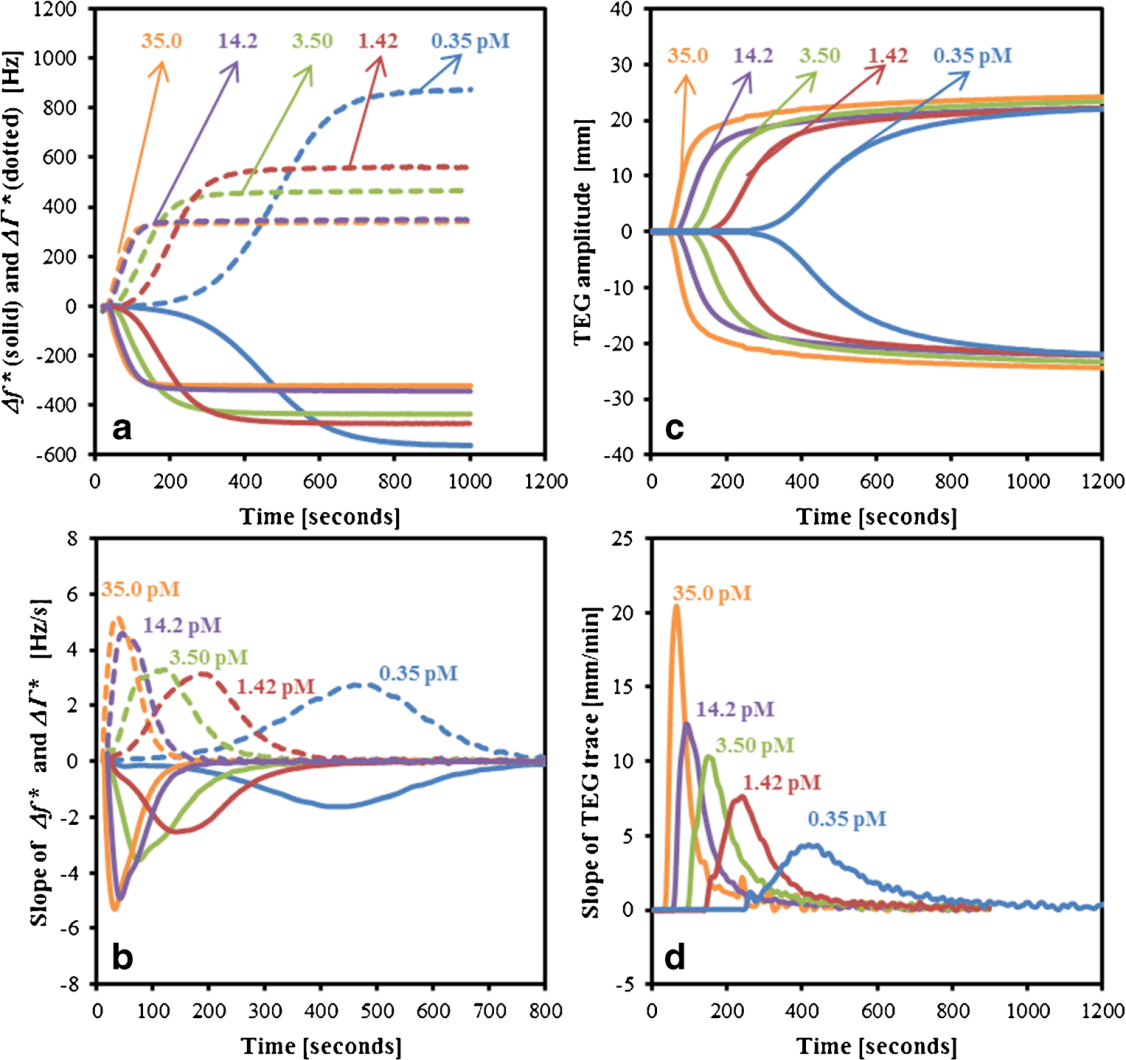
(**A**) Changes in frequency and bandwidth (Δ*f* ^*^ and Δ*Γ* ^*^) for the normal platelet-poor plasma samples activated using TF concentrations of 0.35, 1.42, 3.5, 14.2 and 35 pM. (**B**) Slope of Δ*f* ^*^ and Δ*Γ* ^*^ for the five corresponding concentrations of TF. (**C**) Corresponding TEG traces for the plasma samples activated using different TF concentrations. (**D**) Slope of TEG trace. Increased concentrations of TF resulted in increased onset of clot formation, shorter time to reach maximum slope, greater maximum slope values and lower values of Δ*f* ^*^_max_ and Δ*Γ* ^*^_max_

The activation of the coagulation cascade of citrated PPP was carried out with TF and CaCl_2_ on QCM and TEG. Both QCM and TEG monitor the kinetics of viscoelastic changes occurring during the entire coagulation process. Five concentrations of TF (0.35, 1.42, 3.5, 14.2 and 35 pM) were used to study its effect on the kinetics of clot formation as defined by their measured responses. For QCM, Δf^*^ = (Δ*f*(*t*) − Δ*f*_0_) and ΔΓ^*^ = (Δ*Γ*(*t*) − Δ*Γ*_0_) following activation with various TF concentrations are shown in Fig. 1A, while Fig. 1B shows the first derivative of Δf^*^ and ΔΓ^*^ indicating the rate of clot formation. Figure 1C, D shows the analogous TEG parameters of amplitude and its first derivative, respectively. It is evident that the concentration of TF had a considerable effect on the characteristics of QCM frequency and bandwidth response and the TEG response of amplitude. At lower concentrations of TF, the onset of changes in *f* and *Γ* was increasingly delayed, as were changes to the TEG amplitude. Decreased TF concentration is associated with a delay in the onset of clot formation [24]. It was also seen that the magnitude of both Δf^*^_max_ and ΔΓ^*^_max_ (the maximum change in Δf^*^ and ΔΓ^*^) was dependent on the TF concentration, being greater at lower concentrations. However, final TEG amplitude (MA) was insensitive to the concentration of TF, indicating differences in the interaction of the forming clot with the QCM surface. The dependence of Δf^*^ and ΔΓ^*^ on TF concentration cannot be attributed solely to increased mass attaching to the surface due to a greater affinity of the activated plasma sample [19]. The concentrations of fibrinogen in the tested samples were within the normal range (2.3–3.4 mg/mL). Thus, the differences in the Δf^*^ and ΔΓ^*^ for different TF concentrations are due to differences in the viscoelastic characteristics of the fibrin network coupled to the QCM surface, which was not observed using TEG. TEG and QCM are, in principle, different methods, and the TEG parameter of MA is an indicator of clot strength through a non-linear combination of viscosity and elasticity, which appears to be insensitive to differences in TF concentration-dependent physical properties of the clot.

The amount and rate of thrombin generated during coagulation is known to have a vital influence on the properties of the fibrin clot [4, 24, 25]. The kinetics of clot formation were shown to be dependent on the TF concentration on both QCM and TEG, where a decreased maximum rate of clot formation (MRCF/MRTG) and prolonged time to reach the maximum rate (TMRCF/TMRTG) were observed for decreased TF concentrations. At 0.35 pM of TF, the maximum rate measured on the QCM was 2.75 Hz/s (MRCF-ΔΓ^*^) and the respective time to achieve the maximum rate was 463 s (TMRCF-ΔΓ^*^), while for a concentration of 35 pM, the maximum rate was 5.17 Hz/s (MRCF-ΔΓ^*^) and was achieved in 37 s (TMRCF-ΔΓ^*^). Similarly, on TEG, the measured values for TMRTG and MRTG were 4.3 mm/min and 415 s, respectively, in response to 0.35 pM of TF, while for 35 pM, these were 20.3 mm/min and 64 s, respectively. This suggests that TF concentration affects the quantity and rate of thrombin generated, which also results in characteristically different fibrin clots and not just differences in the rate at which the clot is formed. The differences in the rate of change of Δf^*^ and ΔΓ^*^ (Fig. 1B) are due to the differential contributions from the mass coupled to the QCM surface and the corresponding viscoelastic properties of the clot.

### Comparison of QCM and TEG parameters

**Fig. 2:**
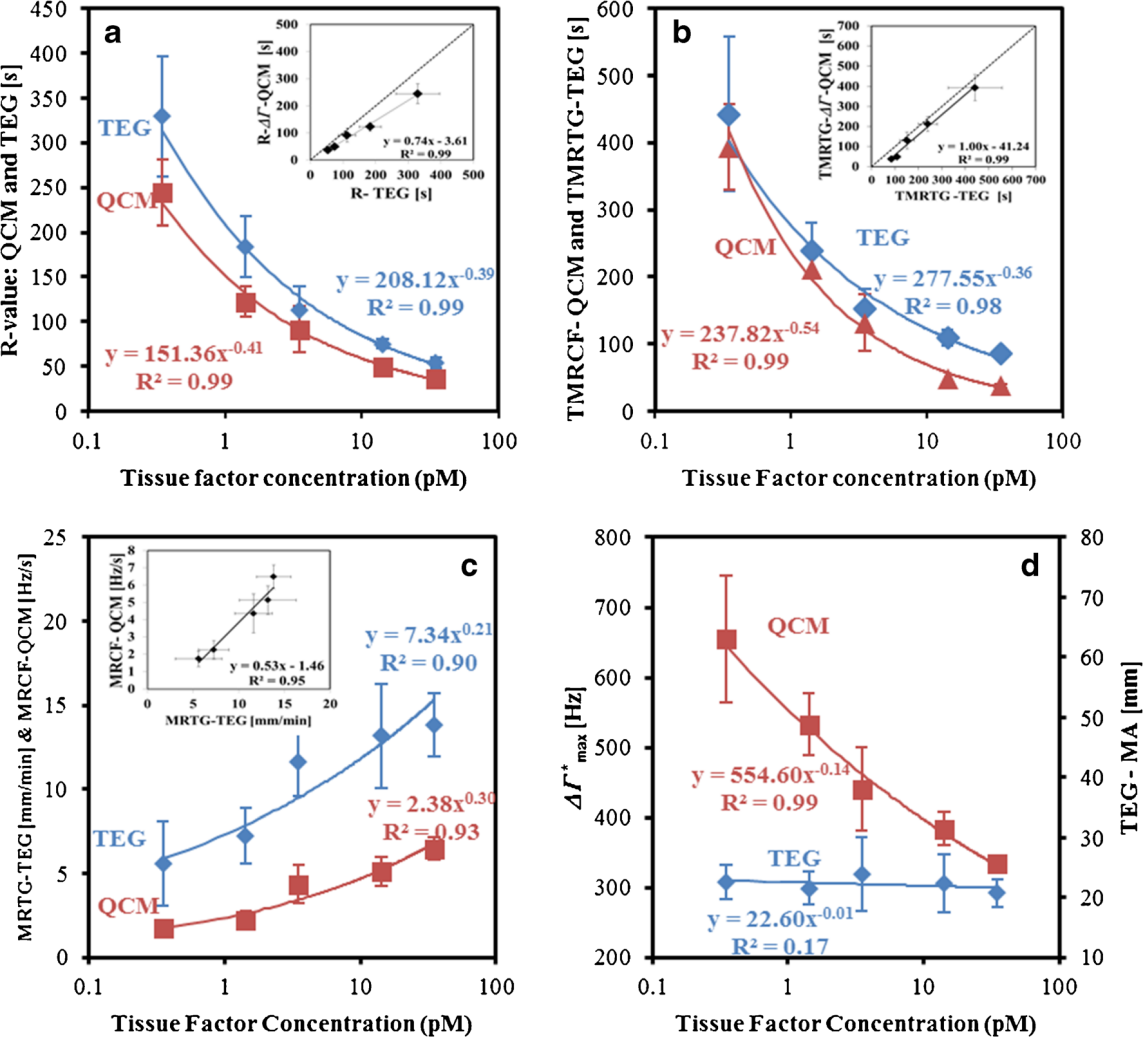
Effect of TF concentrations on the kinetic parameters measured on QCM and TEG. *Insets* show the correlations between TEG and QCM parameters (*n* = 8). (**A**) *R* value (s). (**B**) Time to maximum rate of clot formation on QCM (*TMRCF-QCM*) and time to maximum rate of thrombus generation on TEG (TMRTG-TEG) (s). (**C**) Maximum rate of clot formation on QCM (*MRCF-QCM*) and maximum rate of thrombus generation on TEG (*MRTG-TEG*). (**D**) QCM-Δ*Γ* ^*^_max_ and TEG maximum amplitude (*MA*)

For comparisons between QCM and TEG, only the values for each of the kinetic parameters obtained from the ΔΓ^*^ observations for QCM are presented here as kinetic parameters obtained from Δf^*^ observations (not shown) are analogous and exhibit similar trends to the ΔΓ^*^ observations. To compare the kinetics of the clot formation, the same measurements using identical reagent concentrations were conducted on the TEG instrument. In terms of clotting kinetics, *R*-ΔΓ^*^, TMRCF-ΔΓ^*^ and MRCF-ΔΓ^*^ values correlated well with the TEG measurements of *R*-, TMRTG and MRTG, respectively (Fig. 2A–C). This suggests that the QCM is effective in deriving kinetic parameters and is in good agreement with an established system such as TEG. The time parameters measured on the QCM were all lower than that measured on TEG, which can be attributed to the fact that the QCM measures changes occurring close to the surface (approx. 200 nm) and hence identifies kinetic information sooner than TEG, which requires the forming clot to couple the cup and pin, which are separated by approx. 100 μm. However, analogous parameters that showed a significant difference between QCM and TEG were ΔΓ^*^_max_ (and Δf^*^_max_) and MA. While MA was independent of TF concentration, QCM exhibited a quasi-linear inverse relationship between TF concentration and ΔΓ^*^_max_ (Fig. 2D). It is interesting to note that QCM demonstrated this difference under identical experimental conditions to TEG, suggesting that QCM is indeed measuring a difference in the structure of the formed clot which is not detectable using TEG.

### Image analysis and analysis of physical characteristics of clots formed on QCM

**Fig. 3:**
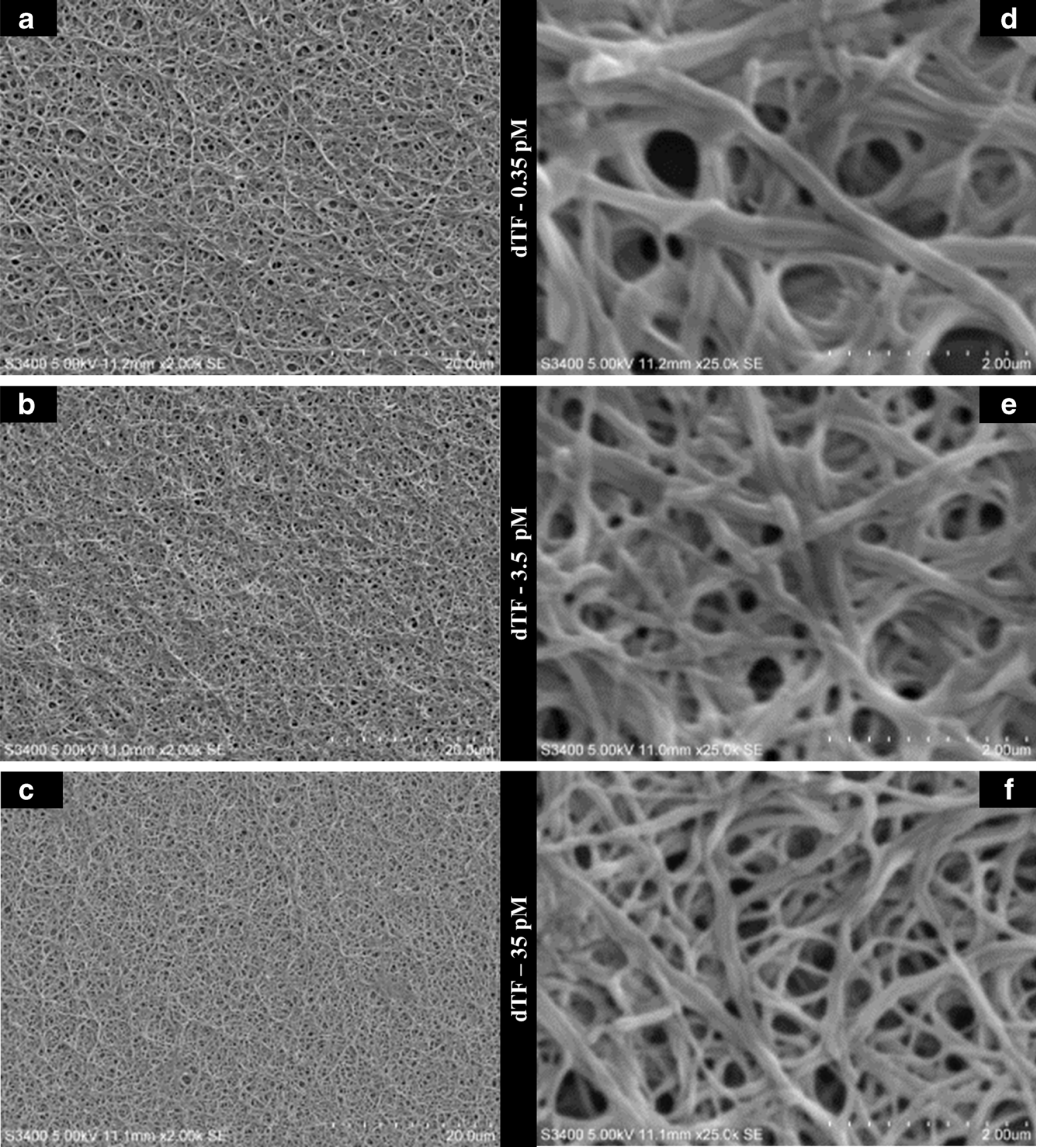
The (**A**–**C**) 20,003 magnification SEM images, used for image analysis, and (**D**–**F**) 250,003 magnification SEM images of clots formed with 0.35, 3.5 and 35 pM of TF, respectively

In order to investigate whether the differences measured in QCM were related to the structure of the formed clot, three of the TF-activated samples (0.35, 3.5 and 35 pM) were imaged at the end of the clot formation process using SEM. Figure 3 shows SEM images of the three samples at 20,003 and 250,003 magnification. Clear differences in clot structure can be seen with the naked eye at higher magnification (Fig. 3D–F), and at least two structural parameters can be observed. These are the fibre density (the number of fibres per unit area) and fibre diameter. It appears that higher TF concentrations make the formed fibrin network denser, but with thinner fibres. The TEG parameter MA has been extensively used to characterise the mechanical property of a formed clot and is widely interpreted as relating to fibrin clot firmness or strength [9, 26] and representing the bulk mechanical properties of the fibrin polymer network and not being related to the properties of individual fibre segments. The results achieved here appear to demonstrate that MA was unable to differentiate between different fibrin networks formed with various TF concentrations (Fig. 2D). Thus, the effect that the rate of clot formation can have on micro-mechanical clot structure was not detected.

Image analysis further confirmed the visual observations of the clot characteristics in Fig. 3 and demonstrated that the average fibrin diameter (F.Diam), fibre density (F.Dens) and clot porosity (F.Por) in the fibrin network were dependent on the rate of clot formation, resulting in final clots with different structural properties (Table 1).

**Table 1:** Results of spectral analysis and modelling parameters of the clots formed with different tissue factor concentrations

TF (pM)	F.Por (a.u.)	F.Dens (μm^−1^)	F.Diam (nm)	*μ*_*t*_ (*n* = 8) (a.u.)	*M*_*t*_ (*n* = 8) (a.u.)	RF (*n* = 8) (a.u.)
0.35	0.518 ± 0.026	1.72 ± 0.09	163 ± 8	1.39 ± 0.21	1.54 ± 0.12	0.91 ± 0.08
3.5	0.525 ± 0.032	1.92 ± 0.12	143 ± 9	0.92 ± 0.12	1.22 ± 0.11	0.75 ± 0.04
35	0.561 ± 0.045	2.12 ± 0.17	118 ± 12	0.67 ± 0.04	0.90 ± 0.01	0.69 ± 0.004

Thus, QCM appears to have a unique advantage over TEG as, in addition to the measurement of clotting time and the kinetics of the clot formation process through characteristic changes in both frequency and bandwidth, these parameters are also dependent on the mass and the viscoelastic properties of the clot interacting with the QCM surface, which can provide meaningful physical information about its structure.

Time courses, Δ*f*(*t*) and Δ*Г*(*t*), as well as MA(*t*) provide information about the kinetics of clot formation, namely rates/slopes of reaction, clotting time and maximum “clot strength”. Moreover, in previous work, it has been shown that the respective final maximum values, Δ*f*_max_ and Δ*Г*_max_, observed when clot formation was complete correlated well with fibrinogen concentration in PPP samples [11, 12]. However, the physical meaning and corresponding direct interpretation of MA(*t*), Δ*f*(*t*) and Δ*Г*(*t*) are challenging. Indeed, in QCM, two measured parameters cannot provide an interpretation of at least three physical processes taking place in the real plasma sample during coagulation: change in viscosity, change in elasticity and fibrin adhesion to the sensor surface. MA is even less informative, being the only measured parameter. In order to derive physical meaning, a mathematical model of “blood component flexibility” has been developed [18]. The model is based on the assumption that serum viscosity does not change during coagulation, which allows for the determination of a normalised effective elasticity (*μ*_*t*_) and normalised effective mass density (*M*_*t*_) of the fibrin adhered to the QCM surface. Another modelling parameter is defined as the ratio of these two parameters, the so-called “rigidity factor” (RF)

1 $$ \mathrm{R}\mathrm{F} = \frac{\mu_t}{M_t} = \frac{a\cdot \left(\varDelta {\varGamma}^2-{c}^2\varDelta {\varGamma}_z^2\right)}{b\cdot \left(\varDelta f-c\varDelta {f}_z\right)} $$

where values Δ*Г*_*z*_ and Δ*f*_*z*_ characterise viscous plasma property and are taken at the time where a plasma sample is just deposited on the sensor surface but before the start of clot formation; *a* and *b* are simple scaling normalisation factors for effective elasticity and effective mass density, respectively (they are set to be *a* = 2.5 × 10^−7^ Hz^−2^ and *b* = 1 × 10^−3^ Hz^−1^ in the present work); *c* is the plasma viscosity correction coefficient defined as *c* = Δ*Г*_*z*_ / Δ*Г*. The equation derivation and detailed explanation can be found in [18]. It has been demonstrated that RF for partial thromboplastin-based activation of PPP was not related to fibrinogen concentration in artificially prepared fibrinogen-spiked samples [18]. Moreover, RF(*t*) was constant throughout the clot formation process and, hence is a qualitative characteristic of the fibrin segments attached to the QCM surface.

Using the described model, RF values were determined for the TF-activated PPP samples described here and investigated as a meaningful physical parameter of clot structure (Table 1). The values derived demonstrate that RF values were significantly different for the three TF concentrations chosen. RF was highest when fibres were shorter, wider and fewer in number as a result of slow clot formation in the presence of low TF concentrations and decreased as fibres became longer, thinner and less numerous due to the more rapid onset of clot formation at higher TF concentrations. Thus, one can use RF as a single derived parameter to infer microscopic structural information about the nature of the formed clot and its origins in a manner not possible with current global coagulation tests. Such information could be useful in studying a range of haemostatic disorders.

## Conclusions

The QCM sensor was found to be capable of monitoring the coagulation kinetics of a plasma sample activated by TF in a manner analogous to thromboelastography. However, QCM was shown to be further capable of detecting qualitative changes in clot structure resulting from differential rates of clot formation at different TF concentrations. This behaviour could not be observed with thromboelastographic parameters. In conjunction with a mathematical model of blood component flexibility, the parameter of rigidity factor could be derived and which relates in physical terms to the viscoelastic properties of the clot. It was demonstrated by visual and image analysis of the clot that rigidity factor could be related to the microscopic structure of the formed clot, which was in turn related to its rate of formation and the TF concentration used. Such a technique could be used to derive important information about the clotting process. In addition, QCM is easy to operate for real-time analysis of blood coagulation and has the potential to be used for point-of-care diagnostics.
